# Kinetics of solvent supported tubule formation of Lotus (*Nelumbo nucifera*) wax on highly oriented pyrolytic graphite (HOPG) investigated by atomic force microscopy

**DOI:** 10.3762/bjnano.9.45

**Published:** 2018-02-07

**Authors:** Sujit Kumar Dora, Kerstin Koch, Wilhelm Barthlott, Klaus Wandelt

**Affiliations:** 1 Institute of Theoretical and Physical Chemistry, University of Bonn, Wegelerstrasse 12, 53115 Bonn, Germany; 2 Rhine-Waal University of Applied Science, Faculty of Live Sciences, Marie-Curie Str. 1, 47533 Kleve, Germany; 3 Nees Institute for Biodiversity of Plants, University of Bonn, Venusbergweg 22, 53115 Bonn, Germany; 4 Institute of Experimental Physics, University of Wroclaw, pl. M. Borna 9, 50-204 Wrocław, Poland

**Keywords:** AFM, crystallization, epicuticular wax, Lotus, *Nelumbo nucifera*, nonacosanol tubules, self-assembly, superhydrophobic

## Abstract

The time dependence of the formation of lotus wax tubules after recrystallization from various chloroform-based solutions on an HOPG surface at room temperature was studied by atomic force microscopy (magnetic AC mode) taking series of consecutive images of the formation process. The growth of the tubules oriented in an upright fashion follows a sequential rodlet→ring→tubule behavior. The influence of a number of factors, e.g., different wax concentration in chloroform, the additional presence of water, or salts [(NH_4_)_2_SO_4_, NH_4_NO_3_] or a mixture of salt/water in the solution on the growth rate and orientation of the tubules is also investigated. Different wax concentrations were found to have no effect on the growth rate or the orientation of tubules in none of the solutions. The presence of water, however, considerably increased the growth rate of tubule formation, while the presence of salt was again found to have no effect on growth rate or orientation of tubules.

## Introduction

The plant cuticle, a cutin matrix embedded and covered by waxes provides a multitasking interface between plant and environment [1]. These waxes are either reside within the cutin layer (intracuticular wax) or deposited over the cutin surface (epicuticular wax) of primary plant organs. Being the first point of contact between plants and environment, the cuticle provides protection against water loss and external environmental stresses. Other important functions include control of transpiration, hydrophobicity, protection of photo synthetic cells, interaction with chemicals and other organisms, providing optical properties etc. [2–9]. Plant waxes are a conglomerate of various long chain (>C_20_) hydrocarbons, aldehydes, ketones, acids, alcohols etc. [10]. Further, cyclic compounds, e.g., pentacyclic triterpenoids or flavonoids as components of cuticular waxes are also reported in literature [11–14]. A comprehensive classification of most types of epicuticular waxes was reported by Barthlott et al. [15]. Additionally, the plant waxes were found to be crystalline in nature as confirmed by a number of experimental techniques, e.g., X-ray powder diffraction (XRD), electron diffraction (ED) and nuclear magnetic resonance (NMR) techniques [16–19].

Out of the vast variety of wax morphologies, tubules constitute one of the most prominent types and can be distinguished into three chemical and morphological groups [20]. They are divided in to tubules containing i) high amounts of secondary alcohol, e.g., nonacosan-10-ol and its homologues; ii) high amounts of alkanediols and iii) high amounts of β-diketones. In this work, tubules of type ii containing predominantly diols [21] are taken in account to understand how different factors affecting self-assembly of these tubules on HOPG.

Koch et al. [21] demonstrated that self-assembly of nonacosan-10-ol tubules resulted in an upright orientation of tubules on HOPG. By employing tapping mode atomic force microscopy (AFM), they observed the continuous growth of tubules after applying a 10 µL droplet (conc. 1.5 mg/mL) onto an HOPG surface. The growth followed a behavior where wax molecules first aggregated to form rodlets which then changed to a circular structure before growing longitudinally in an upright fashion to the surface by the continuous incorporation of wax molecules. The crystallization process was observed up to 4 hours before the increasing height of the tubules led to experimental artifacts. In addition, they also observed an increase in hydrophobicity of the wax structures, demonstrated by an increase in contact angle from 88° to 129° after 14 days of wax recrystallization on HOPG.

A more recent surface scientific approach by Koch et al. [22] demonstrated a spiral growth mechanism of tubule formation. The effect of substrate polarity on tubule growth was also demonstrated by showing upright oriented tubules on HOPG (nonpolar, crystalline) and horizontally oriented tubules on glass (polar, amorphous). They also found that pure nonacosan-10-ol does not form tubules, but that an admixture of 3-4% of corresponding diols is needed for tubules to form. In addition, they also portrayed the effect of chirality by demonstrating tubule formation from the naturally occurring S-enantiomer of nonacosan-ol, whereas synthetic R-enantiomer failed to form such tubules. Temperature change was also found to have an effect on the tubule growth. By evaporating 1 mg of wax onto glass, Koch et al. [22] also demonstrated that at 25 °C tubules do not form whereas at 50 °C tubule formation continues until 7 days resulting in the formation of upright oriented tubules on HOPG. Wax tubule formation over a longer period of time was studied by Niemietz et al. [23], and increasing tubule sizes were found to correlate with an increase of superhydrophobicity of the wax coated surface. The time and temperature related formation of wax tubules has also been used to develop first artificial Lotus leaves for various wetting studies [24].

However, there are still a number of other factors that can affect tubule growth on HOPG. For example, the concentration of wax molecules in the applied solution and the presence of any foreign substances, e.g., water or salts in the wax solution. In plants, the transport of wax molecules from the location of synthesis inside the cells onto the cuticle is discussed as co-transport of the wax components with water evaporation. Evidence for a water co-transport has been given by in-vitro studies with isolated waxes and artificial polyurethan membranes. Neinhuis et al. [25] showed that in the presence of water, waxes were transported through the membranes and reassembled into three dimensional waxes onto the membranes. Studies with hygroscopic salt particles, e.g., ammonium sulphate salts, in contact with leaf waxes showed that waxes move from the leaf surface and grow over the salt particles (Burkhardt et al. [26]). This observed transport of wax was also assigned to a co-transport with water onto the hygroscopic salt particles.

The objective of the present study is to investigate the influence of water and salts on the tubule growth on HOPG further. For this purpose, we have studied the growth rate and morphology of tubules from four different types of chloroform-based solutions as specified in the Experimental section. We demonstrate how these different factors affect the tubule growth, in order to gain greater insight into those factors that can affect the tubule growth and which can subsequently be used to manipulate these wax structures on HOPG, e.g., in order to model specific superhydrophobic surfaces.

## Experimental

The experiments are performed in a similar manner as described in literature [27]. The nonacosan-10-ol wax obtained by extraction of lotus (*Nelumbo nucifera*) leaves with chloroform was obtained from the Nees Institute for Biodiversity of Plants of the Bonn University [18]. This wax was dissolved in four different types of chloroform based solutions: i) pure chloroform containing different wax concentrations (0.2, 0.8, 1.0, 10 mg/mL); ii) a solution of 0.8 mg/mL of wax in water saturated chloroform (solubility of water in chloroform 0.56% at 20 °C); iii) a solution of 0.8 mg/mL of wax in chloroform (dipole moment 1.15 D) saturated with (NH_4_)_2_SO_4_ or NH_4_NO_3_; and iv) a solution of 0.8 mg/mL of wax in chloroform which prior to adding the wax molecules was saturated with a solution of salt in water. The HOPG crystal used in our experiments was bought from SPI suppliers, Germany. The AFM measurements were carried out with a Picoscan AFM (Molecular Imaging, Tempe, AZ, USA) with a PicoScan controller coupled with a MAC Mode controller. The system was used to operate in the MAC Mode employing type I MAC levers under ambient conditions. Type I MAC silicon cantilevers with a length of 90 µm and a typical tip radius of curvature less than 10 nm having a resonant frequency of 75 kHz and a force constant of 3 N/m (NanoAndMore GmbH, Wetzlar, Germany) were used. Appropriate AFM conditions were a scan size of 1–5 µm, a scan rate of 0.5–1 lines/s with an image size of 512 × 512 pixel. A set point near the upper limit was chosen to minimize the interaction between tip and sample. All experiments were performed at room temperature (between 20–24 °C).

For real time observations of wax recrystallization by AFM, a 10 µL droplet of the respective wax solution was applied onto the HOPG surface. The chloroform takes ≈30 seconds to evaporate from the surface leaving the wax attached to the substrate. The HOPG substrate was then fixed to the stainless steel AFM base plate using a metal clip, so that the first image acquisition started typically 8–10 minutes after application of the wax solution, i.e., long after the chloroform evaporation. AFM images were taken consecutively from the same substrate area over a period of several hours applying a constant scan rate (as denoted in the respective figure captions). Height and length measurements of wax crystals were made with the program WSxM (Version 3.0; Nanotec Electronica, Madrid, Spain).

## Results

In this section we describe the formation of the wax tubules as a function of time. Tubules oriented in an upright fashion similar to those found by Koch et al. [21] on HOPG were also observed in this case. Here, however, the principle findings of wax growth are presented for differently concentrated wax solutions as well as under the influence of foreign additives, e.g., salt, water or both.

### Effect of concentration on tubule growth from pure chloroform

In order to determine changes in the wax recrystallization as a function of the molecular concentration in solution, we have used solutions of varying concentration, e.g., a very low concentration (0.2 mg/mL), intermediate concentration (0.8 and 1 mg/mL) and a very high concentration (10 mg/mL) of lotus wax in pure chloroform.

**Figure 1:**
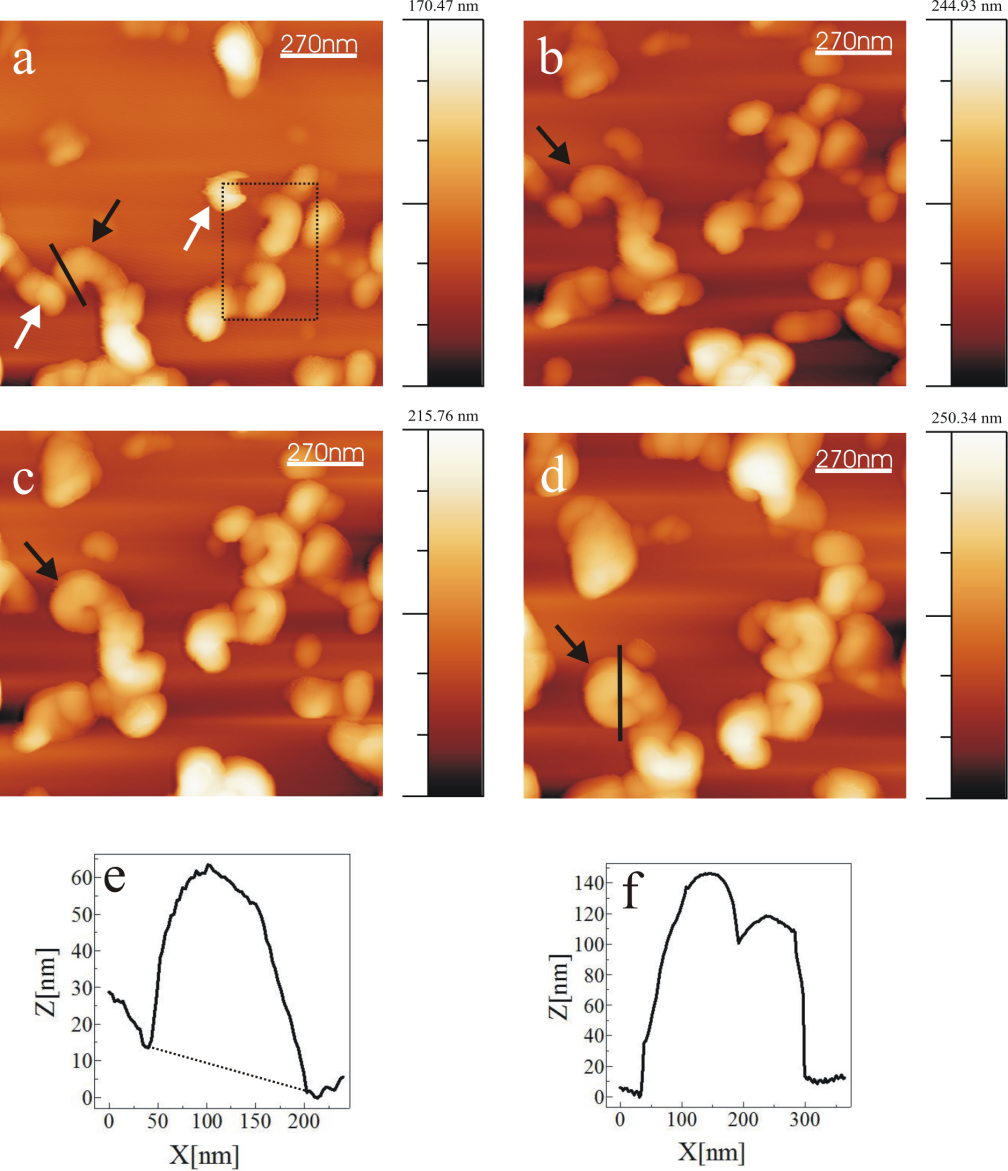
Consecutive AFM images of Lotus (*Nelumbo nucifera*) wax tubule recrystallization on HOPG, taken between 21 min and 215 min after applying a solution of 0.8 mg/mL wax in pure chloroform to the substrate. The black arrow points to a growing tubule in an upright fashion on the substrate. The average time period of completion of tubules is ≈4 h. The growth of the tubules starts from rodlets (a: 21 min, black arrow) and proceeds to curved rodlets (b,c: 43, 150 min, black arrow) before finally forming tubules (d: 215 min) as shown by the arrows. For the dashed rectangle in panel a see Figure 8 and text. Panel e) and f) show the height profiles of (e) the initial rodlet in panel a) and (f) the final tubule in panel d) along the black lines shown in panel a) and d), respectively. Image size = 1*.*35 × 1*.*35 µm, scan rate = 0.796 Hz, 512 lines/image. All images shown in this work include an intensity/height scale on the right side.

**Figure 2:**
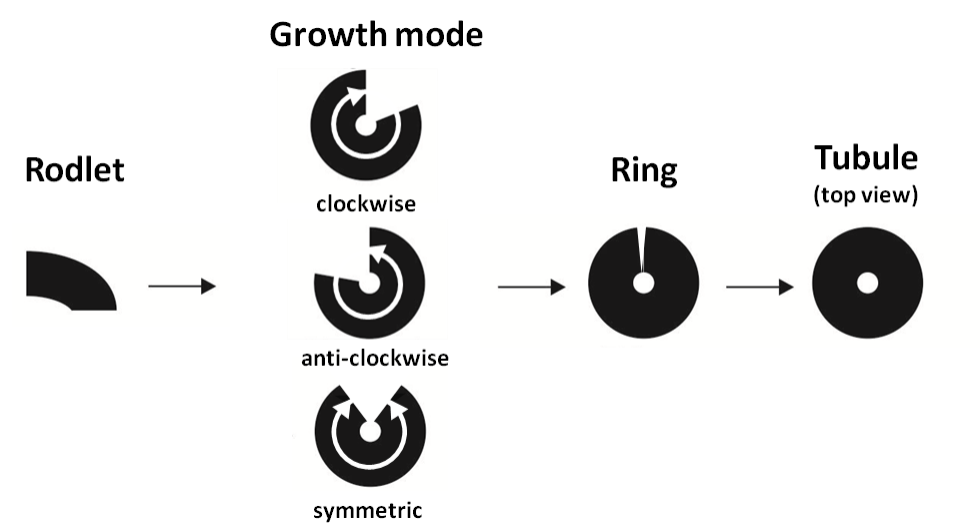
Schematic drawing showing the different stages and modes of tubule formation.

A series of AFM images shown in Figure 1 demonstrate tubule growth after applying a 10 µL droplet of 0.8 mg/mL wax solution onto the HOPG surface. A video comprising a whole series of images is available online (Video 1, Supporting Information File 1). Chloroform being a highly volatile solvent evaporated very quickly from the HOPG substrate resulting in the formation of an inhomogeneous circular pattern of the growing wax. This phenomenon is widely known as “coffee drop effect” (Li et al. [28]). To be consistent with our measurement, all the images and results presented here refer to the central part of the recrystallization area where a more homogeneous wax distribution was found. As shown in Figure 1a, wax aggregates in the form of rodlets were formed in the first 20 min of crystallization. The term rodlets is used here as previous investigations by Koch et al. [21] demonstrated that for these structures the aspect ratio (height to thickness) was much smaller than for waxes called ribbons. The terms height and thickness refer to the vertical distance along Z direction and basal wall thickness of rodlets along X, Y plane respectively. Continuous observation of the crystal growth (see black arrows in Figure 1) shows that the rodlets start to form curved structures by continuous addition of new mass to the growth end. The average time period for the completion of a rodlet structure (as sketched in Figure 2) formation was ≈215 min. (We use the term "completed/final" or "completion" here for the stage when any further measured growth has become smaller than ≈5%.) For the exact acquisition time of individual images the reader is referred to the corresponding figure captions. The average thickness of a rodlet (Figure 1a, black line) and the height of a completed tubule (Figure 1d, black line) is ≈40 nm and ≈110 nm respectively. Heights were measured at the growing end of the tubule (shown by black lines in Figure 1a and 1d) and height profiles are shown in Figure 1e (corresponds to rodlet in Figure 1a) and Figure 1f (corresponds to tubule in Figure 1d). It is also important to note that whereas tubular structures start to grow in height, non-tubular structures start to dissolve during the same time. For example, structures marked by white arrows in Figure 1a have dissolved in Figure 1d. This observation points to the operation of the Ostwald-ripening process. The full width at half maximum (FWHM) height of the profile in Figure 1d is ≈110 nm, and may be regarded as the "wall-thickness" of a final tubule. Accordingly, FWHM of the profile in Figure 1f is ≈250 nm and the dip in the profile f) appears, thus, consistent with the formation of the central hole of the growing tubule.

**Figure 3:**
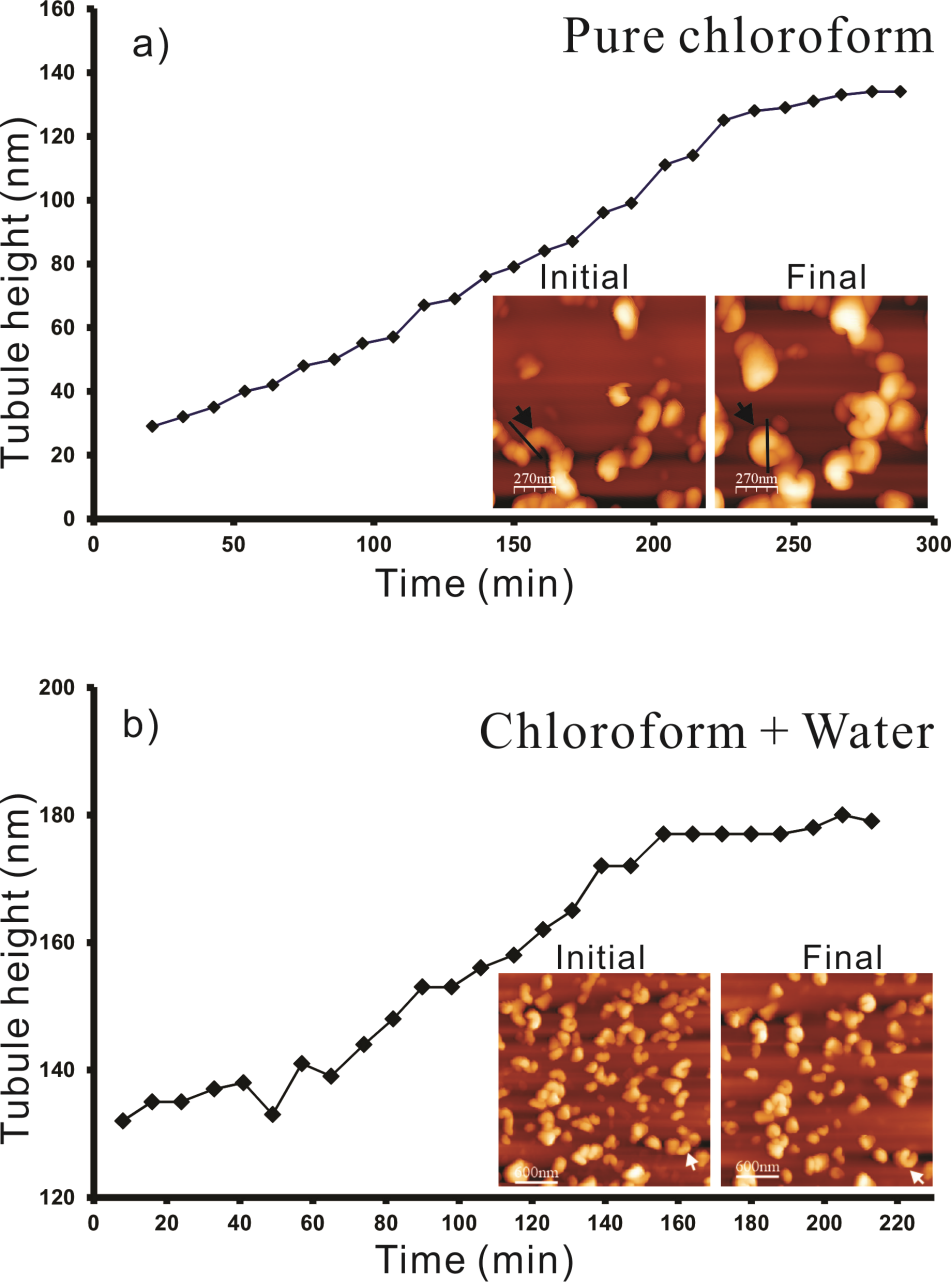
a) Plot of the average height (nm) of Lotus (*Nelumbo nucifera*) wax tubules vs time (min) grown on HOPG from pure chloroform for all the images of Video 1, Supporting Information File 1 (partially shown in Figure 1). (b) Plot of the average height (nm) of Lotus wax tubules vs time (min) grown on HOPG from water saturated chloroform for all images of Video 5, Supporting Information File 2 (partially shown in Figure 7). Note the significantly faster growth rate in the presence of water (see text).

A plot of the average height of the tubular structures versus time for all the images of Video 1, Supporting Information File 1 (partially shown in Figure 1) starting from the initial rodlets to the completed tubules is plotted in Figure 3a. It is important to note that the height measurements are done at the growth end of the rodlets marked by the black lines on images in Figure 3a (see inset, and Figure 1a). Also the final heights of the tubules do not correspond to that of the final tubules but only to that in the last possible AFM image before image artifacts started to occur. According to Figure 3a a progressive increase in height of rodlets was observed until after ≈250 min the growth approaches "completion". According to Figure 3a the final tubule height reaches ≈130 nm after ≈250 min. This results in an average growth rate of roughly 0.5 nm/min (scan rate 0.796 Hz, 512 lines/image). This growth rate*,* of course*,* is an average value for the entire time of tubule growth after the application of the solution. The initial growth rate (prior to imaging) is obviously much faster because the rodlet in Figure 1a marked by the black arrow has already reached a height of ≈40 nm after 21 min indicating a growth rate of 2 nm/min. The initial growth until 20 min could not be plotted as there is always a certain delay in measurement due to cantilever approach time in AFM. This initial growth rate turned out to be independent of the concentration of the applied solution of wax in pure chloroform, so that no further plots need to be shown here.

**Figure 4:**
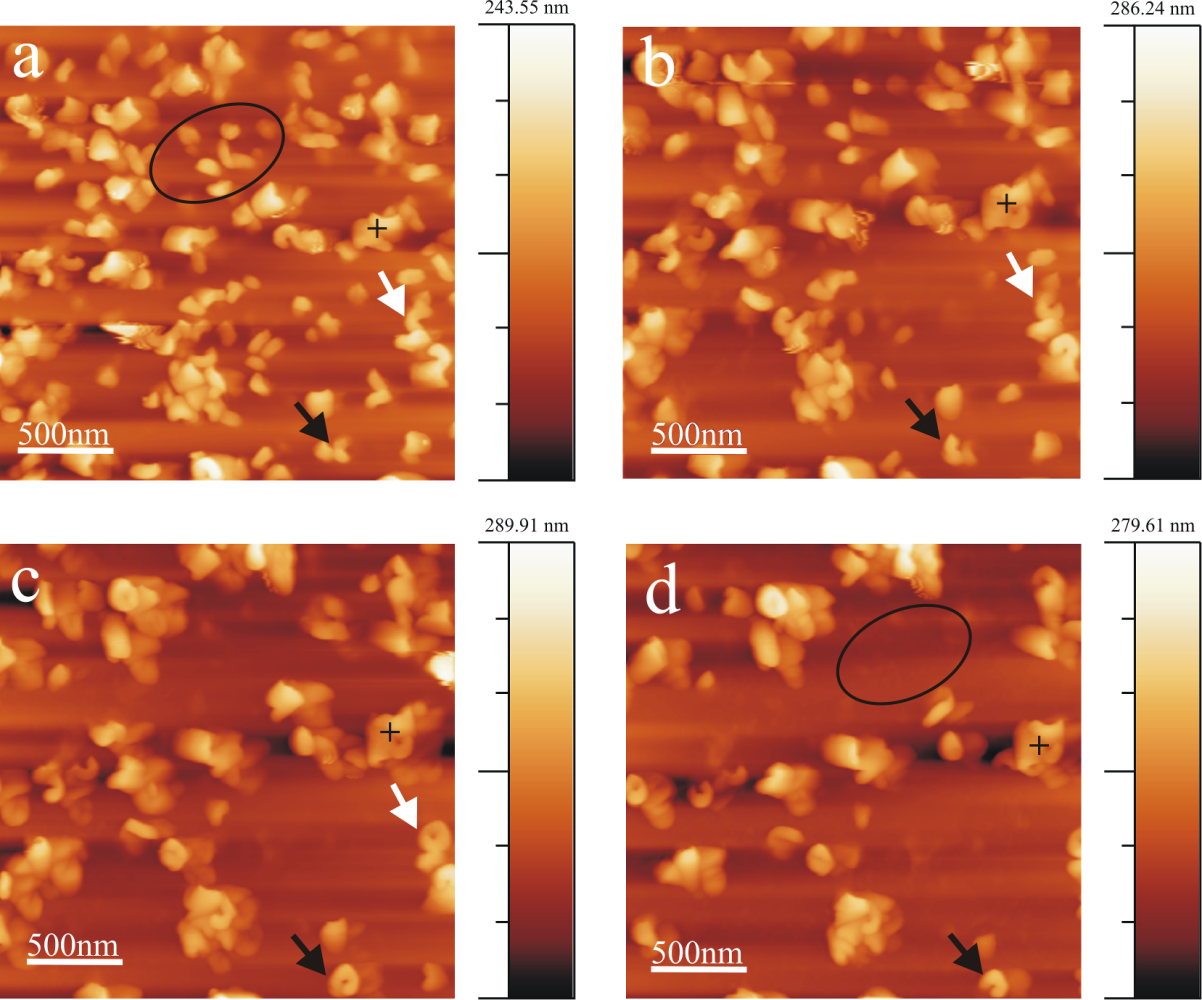
Consecutive AFM images showing Lotus (*Nelumbo nucifera*) wax tubule growth and dissolution on HOPG, registered between ≈33 min and 317 min after applying a 1 mg/mL wax solution (in pure chloroform) to the substrate. The cross marks a stable structure. The black and white arrows point to growing rings, the dashed oval indicates a region of dissolving structures from Panel b to Panel d. Also a completely formed tubule undergoes dissolution (compare Panel c and Panel d, 317 min, black arrow). Average time period of tubule completion is ≈4 h. The growth starts from rodlets (Panel a, 33 min) to form curved rodlets (Panel b, 133 min) before finally forming complete tubules (Panel c, 217 min). Image size = 2.5 × 2.5 µm, scan rate = 0.512 Hz, 512 lines/image.

Another series of AFM images demonstrating both tubule growth and dissolution after applying a 10 µL droplet of 1 mg/mL wax solution onto HOPG is presented in Figure 4 (Video 2, Supporting Information File 3). The growth pattern follows the same rodlet→ring→tubule growth behavior as that observed for the 0.8 mg/mL wax solution (see Figure 1). Thereby the tubule growth can occur either in anticlockwise (white arrow) or in clockwise direction (black arrow). The average time period of formation of a complete tubule is ≈220 min in this case (for the exact timing the reader is referred to the figure captions). During this period also the dissolution of some previously formed but obviously unstable structures are observed, for instance within the oval surface region marked in Figure 4a and 4d. The average heights of the rodlets in Figure 4a are ≈30 nm (black arrow) and 60 nm (white arrow) whereas the height of the completely formed tubules in Figure 3c is ≈110 nm for both tubules. Heights are measured at the growth end of the rodlets similarly as explained above for Figure 1.

**Figure 5:**
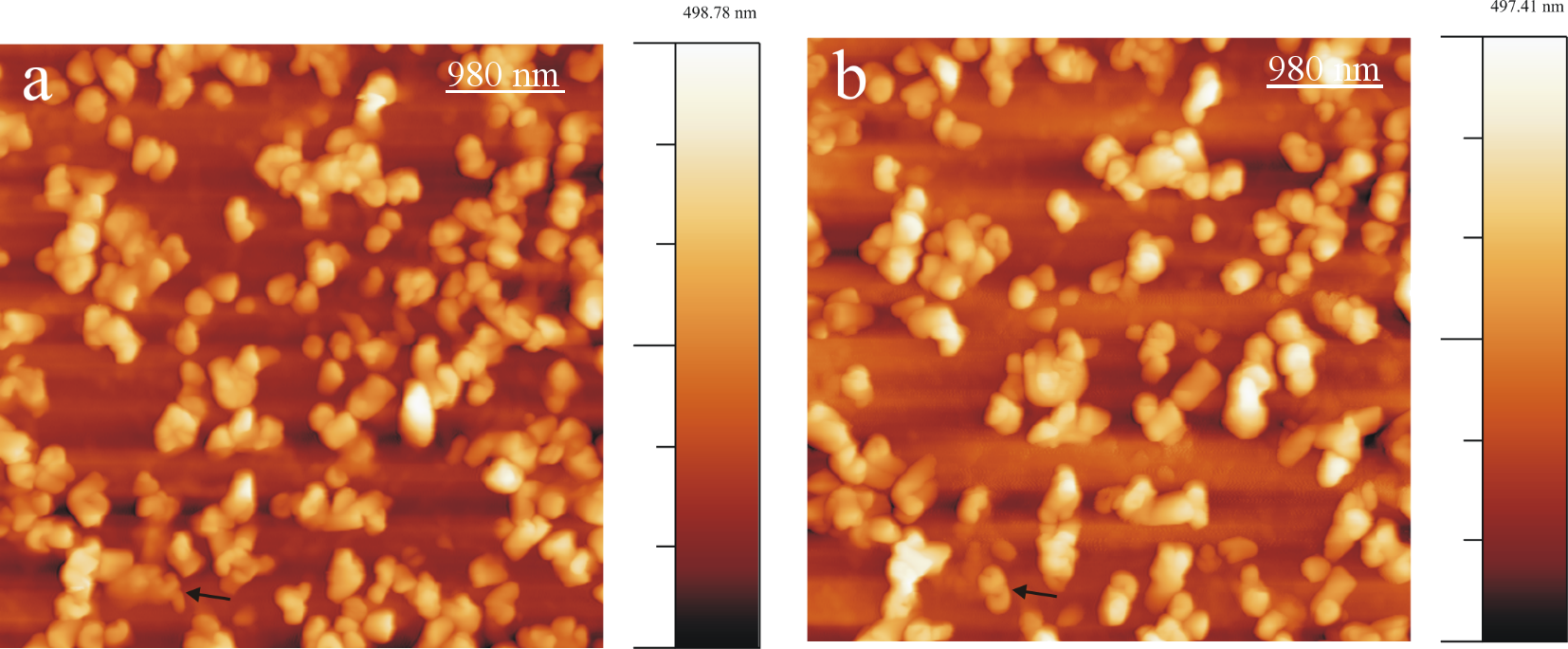
AFM images of Lotus (*Nelumbo nucifera*) wax structures on HOPG after applying a 10 mg/mL wax solution (chloroform solvent) to the substrate. Rodlets grow both clockwise and anticlockwise from the same location to form rings (Panel a, ≈126 min and Panel b, ≈232 min). The average time period of tubule formation is ≈4 h. Image size = 4.9 × 4.9 µm, scan rate = 0.512 Hz, 512 lines/image.

In order to find out whether the nature of the substrate has any effect on the upright orientation of the tubules on HOPG, we used a higher wax concentration, i.e., 10 mg/mL of wax in solution. At such higher concentration, the rapid evaporation of the chloroform suggests the initial formation of a disordered basal wax film being the reservoir for the further long-term growth process. Thus, the tubules eventually grow out of this film rarther than on the HOPG surface. Yet, although the total number of tubules seems to decrease, tubules oriented in an upright fashion are still found at such high concentrations. Figure 5 shows two AFM images of wax crystallization at different time intervals after applying a droplet of 10 µL of a 10 mg/mL wax solution in chloroform onto HOPG (image series available as Video 3, Supporting Information File 4). The black arrow points to two rodlets, one growing clockwise while the other one grows anticlockwise near the same position (for details see Video 3, Supporting Information File 4). The growth behavior follows a similar pattern to that observed for the lower concentrations described above and the average time period of tubule "completion" is again ≈4 h.

**Figure 6:**
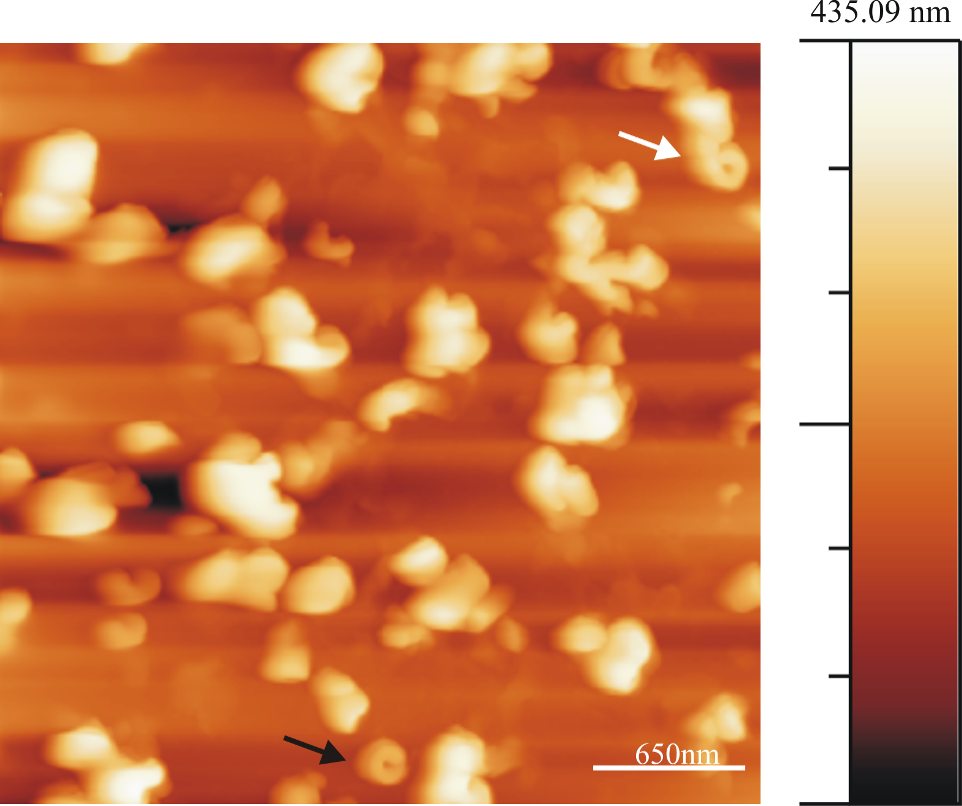
AFM image of Lotus (*Nelumbo nucifera*) wax tubule growth on HOPG after applying 0.2 mg/mL wax solution (chloroform solvent) to the substrate. Even at this very low wax concentration in solution the average time period of complete tubule formation is ≈4 h. Image size = 3.25 × 3.25 µm, scan rate = 0.519 Hz, 512 lines/image.

In turn, in order to gain insight whether a minimum amount of wax is needed for tubule formation we made also one test using a wax concentration of only 0.2 mg/mL in chloroform. However, as shown in Figure 6 tubules still form on HOPG with this low concentration of wax molecules (arrows). The whole sequence of images as video is available online (see Video 4, Supporting Information File 5). The growth pattern resembles completely the one as described above for higher concentration and the average time period of tubule completion is again ≈4 h. It has not been tested here, but appears possible, that tubules can still be formed on HOPG with concentrations lower than 0.2 mg/mL.

### Wax growth in the presence of water

**Figure 7:**
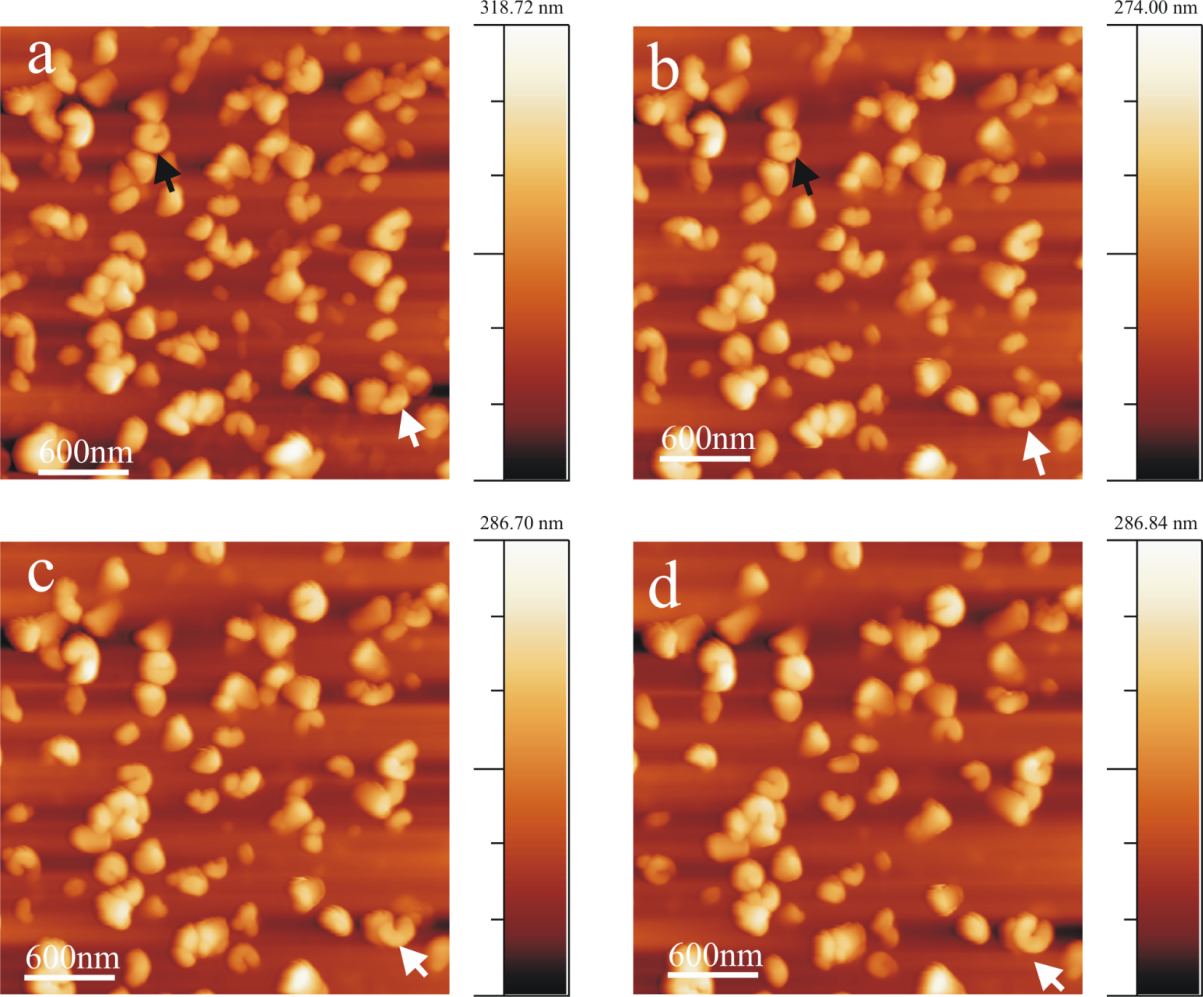
Consecutive AFM images of Lotus (*Nelumbo nucifera*) wax tubule growth on HOPG, taken between 16 min and 131 min after applying a 0.8 mg/mL wax solution (in water saturated chloroform solvent) to the substrate. The black arrow shows an upright grown tubule on the substrate right from the beginning. The average time period of tubule completion is ≈2 h which is only half of the time needed in the absence of water. The growth of the tubules starts again from rodlets (Panel a, 16 min) via curved rodlets (Panel b and c, 49, 90 min) as shown by arrows, before finally forming the complete tubules (Panel d, 131 min). Image size = 3 × 3 µm, scan rate = 1.04 Hz, 512 lines/image.

As mentioned in the introduction it seems rather probable that water plays an important role in the natural formation process of the epicuticular wax layer. This role may not only be that of an inert transport medium. Due to their capability of forming hydrogen-bridge bonds with functional groups of the wax molecules water molecules could even have an influence on the structure of the grown wax objects. Therefore, in order to investigate the influence of water on the wax crystallization process we have studied wax growth from molecules dissolved in chloroform saturated with water, and made two general observations: First, the same successive rodlet→ring→tubule growth sequence is observed; and second, the average time period of tubule "completion" is significantly reduced, namely (in the presence of water) to almost half the time measured in pure chloroform. Figure 7 shows a series of AFM images taken after applying a 10 µL droplet of a 0.8 mg/mL wax solution in water saturated chloroform onto HOPG. The tubule growth follows the same pattern as in the absence of water as described above. A whole image sequence as video is available as supplement material (Video 5, Supporting InformationFile 5). The average time period of tubule completion is only 2 h, i.e., the growth rate in the presence of water is twice as fast as that observed with water-free chloroform. Figure 7a which is taken 16 min after deposition of the solution shows again that rodlets are precursors of tubules which then change to curved structures (see white arrow in Figure 7b and 7c, taken after 49 min and 90 min, respectively) and finally approaching the formation of a complete ring (Figure 7d, taken after 123 min) by the continuous incorporation of wax molecules at the growth sites. In addition, completed tubular structures are already observed at an earlier stage of the experiments (black arrow, Figure 7a and 7b). This is quite different from the situation in the absence of water, where no tubules were found so early (see Figure 1a). This indicates an increased growth kinetics in the presence of water.

A plot of the average height of the tubular structures versus time for all the images of Video 5, Supporting Information File 2 (partially shown in Figure 7) starting from rodlets to complete tubules is plotted in Figure 3b. It is important to note that the height measurements in Figure 7a–d are done at the growth end of the rodlets as exemplified by the black lines in the images in Figure 1a and Figure 1d (and the inset in Figure 3a). Again, the final heights of the tubules plotted in Figure 3b do not yet correspond to the completed tubules but to the last images of the AFM measurements before imaging artifacts occurred. As seen in Figure 3b, 160 min after application of the solution (time = 0 min) to the HOPG surface the measured height of the tubules reaches ≈175 nm, resulting in an average growth rate of roughly 1 nm/min (scan rate of 1.04 Hz, 512 lines/image). As in the water-free case described above the actual growth rate is not constant. For example, in Figure 3b, the rodlet height was already ≈130 nm after 10 min indicating an initial growth rate of ≈13 nm/min in the beginning, i.e., before the actual measurement. Between the onset of measurement and "completion" of the tubules the growth rate was significantly lower, namely ≈0.3 nm/min between 10 min and 160 min and then slowed to <5% of this value as mentioned above. The initial growth prior to measurement could again not be followed due to the cantilever approach time in AFM. Although the slopes in Figure 3a and Figure 3b are almost the same, the initial tubule height in Figure 3b of 130 nm after the first 8 min before image acquisition is about 8 times higher than the rodlet height of only 40 nm after 20 min in Figure 3a in the absence of water. Thus, tubule growth is much faster in the initial stage when water is in the solvent chloroform. However in both cases, without and with water, the growth slows down pointing to a size limitation of the tubules.

### Wax tubule growth in the presence of salts

**Figure 8:**
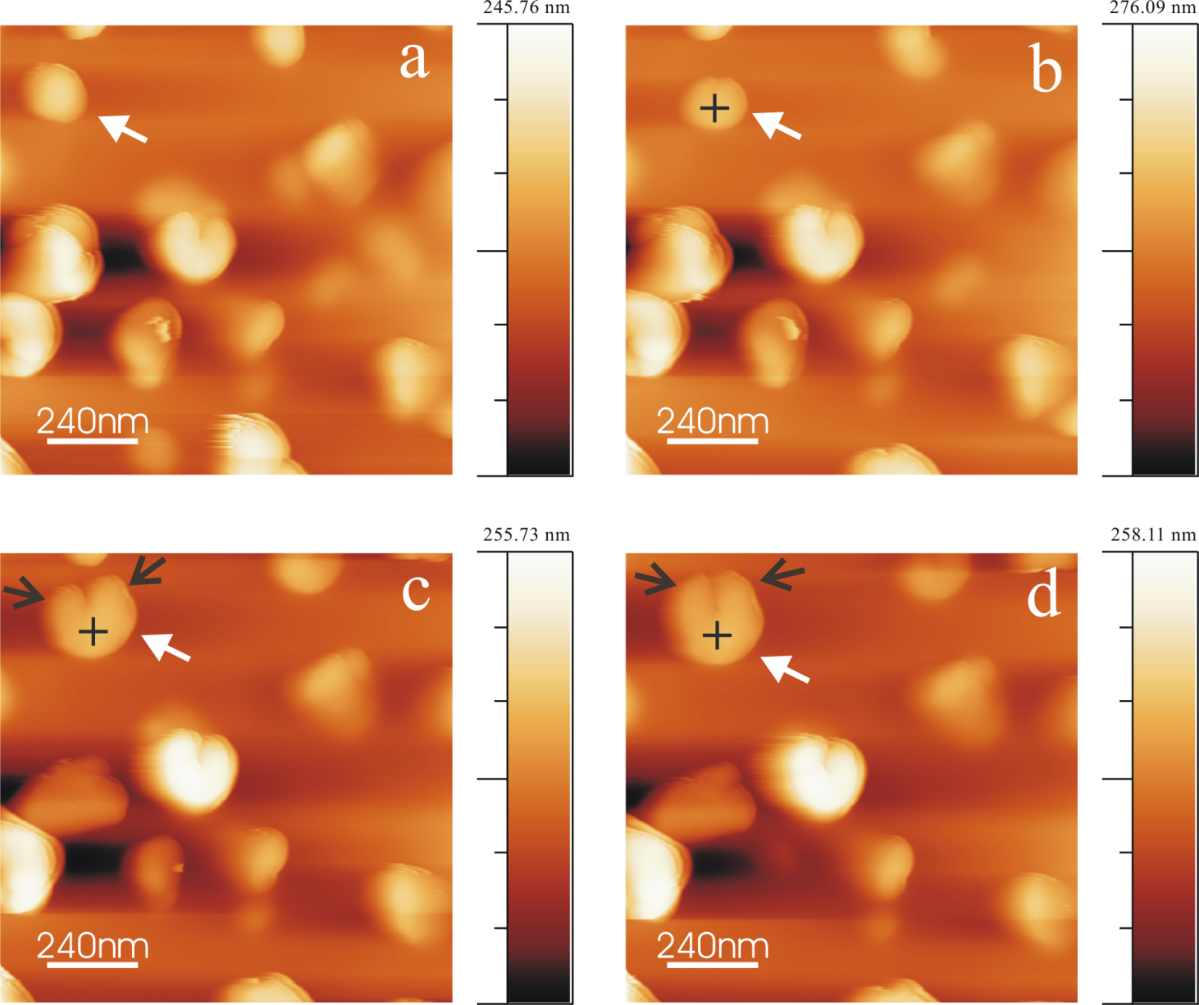
Consecutive AFM images of Lotus (*Nelumbo nucifera*) wax tubule growth on HOPG, taken between 41 min and 213 min after applying a 0.8 mg/mL wax solution (in (NH_4_)_2_SO_4_ saturated chloroform solvent) to the substrate. The white arrow points to a wax feature whose center is marked by the black cross. Note that this feature grows to the left and right side (black arrows). The average time period of forming complete tubules is ≈4 h like from pure chloroform (without salt). The growth of the tubules starts again from rodlets (Panel a, 41 min) via curved rodlets (Panel b and c, 90, 156 min) to finally form complete tubules (Panel d, 213 min). Image size = 1.2 × 1.2 µm, scan rate = 1.04 Hz, 512 lines/image.

**Figure 9:**
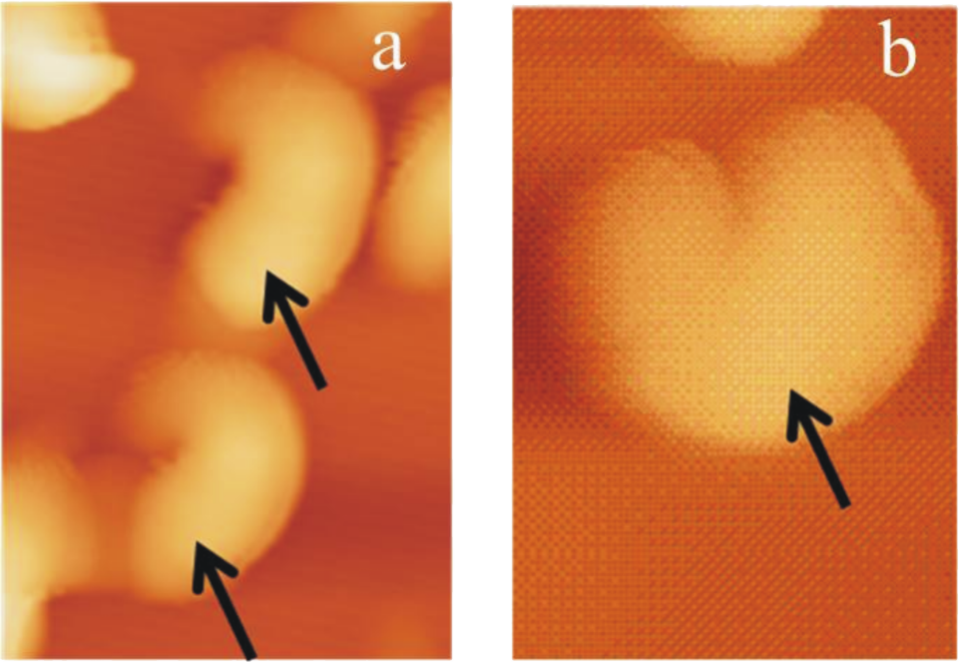
Comparison of the two wax features within the dashed rectangle in Figure 1a with the marked structure in Figure 8d. The black arrows point to the regime of highest intensity, i.e., largest thickness, suggesting unidirectional growth in a) and bidirectional growth in b).

The saturation of (water-free) chloroform with salt does not seem to alter the growth rate or orientation of tubule formation. The average time period of tubule completion is ≈4 h and is comparable to that in the absence of salts or water. Figure 8 shows a series of AFM images (Video 6, Supporting Information File 6) demonstrating tubule formation after applying a 10 µL droplet of a (NH_4_)_2_SO_4_ saturated chloroform solution containing 0.8 mg/mL wax to the HOPG surface. The average time period for tubule completion is ≈4 h (exact time periods are given in the figure captions). The tubule growth follows the usual rodlet→ring→tubule formation behavior. However, also a different type of growth behavior seems to become obvious here. As shown in Figure 8 and enlarged in Figure 9, tubule growth may also continue by the attachment of wax molecules on both ends of the initial rodlet thereby showing a bidirectional growth (see growth of top left tubule in Video 6, Supporting Information File 1). The black cross in Figure 8b–d marks the position of the center of the wax feature marked by the white arrow in Figure 8b. It is obvious from Figure 8c and Figure 8d that this feature grows on both sides of the cross (black arrows). Figure 9 compares a) growing rodlets from Figure 1a with b) the marked rodlet in Figure 8. While both curved rodlets in Figure 9a are asymmetric, i.e., one end (black arrow) is more intense than the other end, the curved rodlet in Figure 9b is most intense in the middle and weakens at both ends. Since this type of bidirectional growth, however, was observed very rarely, i.e., in this particular experiment, it is yet unclear whether any general conclusion about a deviating mechanism for tubule growth in the presence of salt can be drawn, but we did not want to withhold the description of this observation. Ultimately, the experiments with NH_4_NO_3_ saturated (water-free) chloroform solution also resulted in the formation of upright oriented tubules with the same time behavior as in pure chloroform and, hence, are therefore not discussed further.

### Wax tubule growth in the presence of salt/water

**Figure 10:**
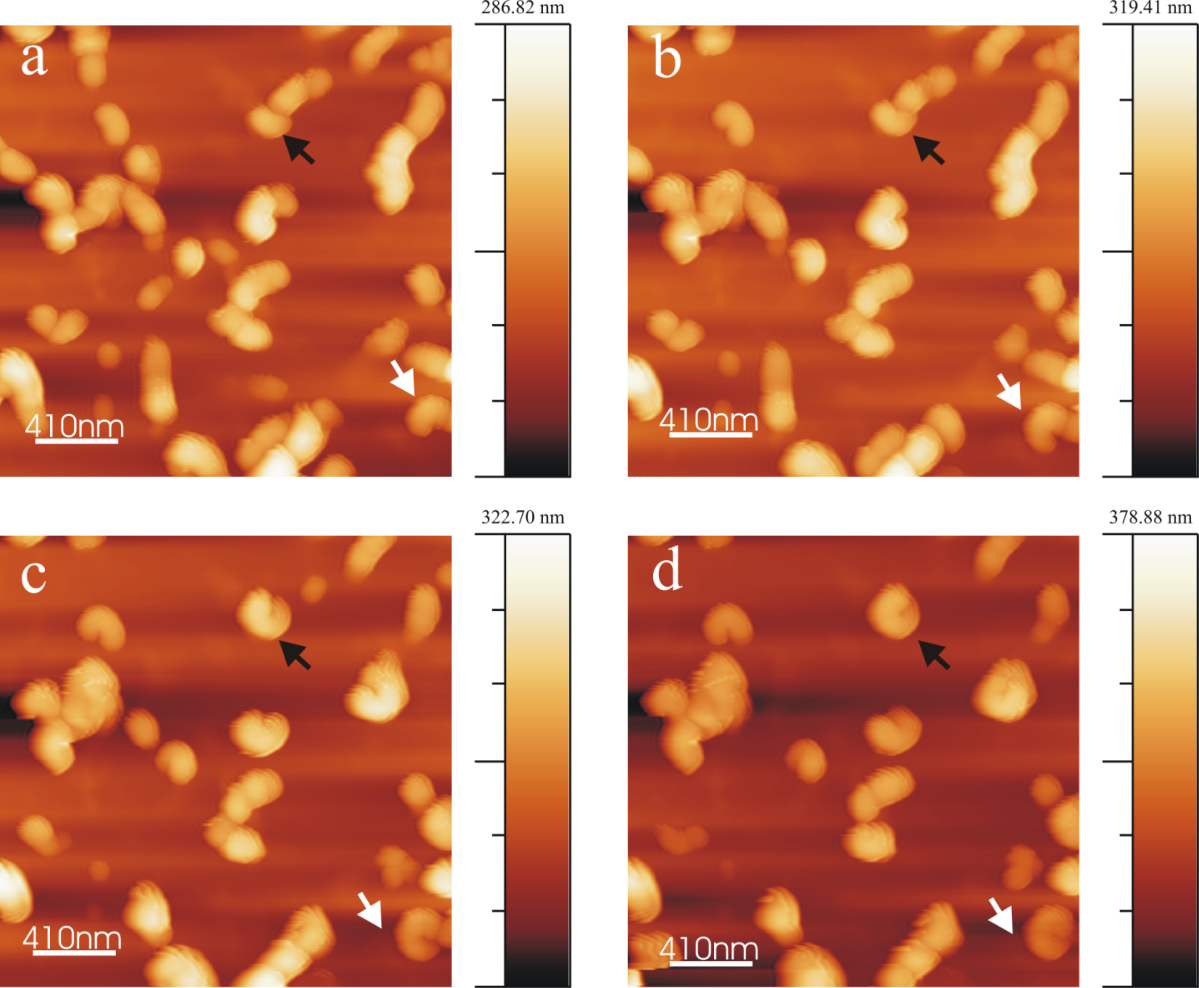
Consecutive AFM images of Lotus (*Nelumbo nucifera*) wax tubule growth on HOPG, taken between 16 min and 115 min after applying a 0.8 mg/mL wax solution (in water/(NH_4_)_2_SO_4_ saturated chloroform solvent) to the substrate. The arrows point to the growing tubules oriented in an upright fashion on the substrate. The average time period of tubule completion is ≈2 hour which is considerably lower than in the absence of water (see text). The growth of the tubules starts again from rodlets (Panel a, 16 min) to form curved rodlets (Panel b and c, 41, 82 min) and finally complete tubules after 115 min (Panel d). Image size = 2.05 × 2.05 µm, scan rate = 1.04 Hz, 512 lines/image.

Tubule growth from chloroform saturated with both salt and water is comparable to the tubule growth in the presence of water only, i.e., again the upright oriented tubules are completed after ≈2 h. Figure 10 shows AFM images after applying a 10 µL droplet of 0.8 mg/mL wax solution in (NH_4_)_2_SO_4_/water saturated chloroform onto HOPG (Video 7, Supporting Information File 7). As observed in Figure 10a, rodlets (marked by arrows) are again the precursors from which tubules are finally formed (Figure 10d). Both clockwise (black arrow) and anticlockwise (white arrow) growth of tubules, i.e., one end appears more intense than the other, is observed. The average height of the rodlets (Figure 10a) and the complete tubules (Figure 10d) are ≈90 nm and ≈160 nm (black and white arrows), respectively. It shall again be noted that tubule growth in the presence of water/NH_4_NO_3_ (figure not shown) follows the same behavior; the growth rate as well as tubule orientation are the same as in the cases of water- and (NH_4_)_2_SO_4_/water-saturated chloroform, i.e., the presence and nature of these anions has no influence, only the presence of water.

## Discussion

After application of the wax molecules from pure chloroform solution onto the HOPG surface at room temperature (20–24 °C) two important observations were made. Firstly, the average time period of tubule "completion" is ≈4 h irrespective of the concentration of the wax molecules in the solution used, i.e., the growth rate is independent of the concentration in solution. Secondly, the tubules are oriented in upright direction to the surface irrespective of whether they grow from a low, medium or very high concentration solution, i.e., the characteristic final surface morphology is also independent of the concentration in solution.

The first observation suggests that the growth process follows at least a two-step mechanism:

[1]
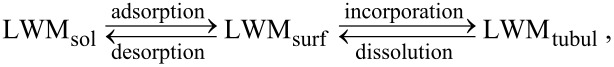


where LWM_sol_ denotes the concentration of dissolved "Lotus wax molecules" in chloroform, LWM_surf_ corresponds to the concentration of LWM molecules adsorbed on the surface, and LWM_tubul_ refers to molecules incorporated into growing or completed tubules. At least the second step (but possibly also the first) is reversible, because the dissolution of initially formed rodlets was observed (see Figure 4). The fact, that the growth rate of tubules is independent of LWM_sol_ means that the adsorption step is very fast, and there are always sufficient molecules adsorbed on the surface not to retard the following slower incorporation process, the latter most likely being dominated by diffusion of the molecules across the surface to their final site of incorporation in a wax tubule. Thus, besides the concentration of adsorbed molecules also their surface mobility to reach the incorporation site matters.

One factor to increase the mobility is temperature. Koch et al. [22] have, indeed, demonstrated that in the *absence* of a wax dissolving liquid, when wax molecules were deposited by vapor deposition onto an HOPG surface, tubules were rarely formed when the substrate was held at 25 °C; only at temperatures 25 °C < *T* < 50 °C was tubule growth observed. In turn, since all experiments in the present work were done at fixed temperature (20–24 °C) it must be the presence of the solvent (chloroform) which increases the surface mobility of the molecules. This possibility was already discussed in Koch et al. [21]. Even though most of the chloroform evaporates instantaneously from the surface (within 30 s) compared to the duration of the following growth of tubules, leaving solid wax behind, there is a chance that some solvent molecules are incorporated between the wax molecules, whose evaporation rate is much slower. These solvent molecules may support the diffusion of the wax molecules.

As to the second observation, namely the independence of the final orientation/morphology of the tubules on the surface, the independence of the growth rate on the concentration in the bulk solution is a first hint: A constant and, in particular, slow growth rate favors the equilibrium structure.

Extensive amount of research is carried out for studying the epitaxial growth of organic molecules with aliphatic chains on HOPG which occurs due to the perfectly-matched interatomic distances in the molecules and the HOPG substrate. Watel and coworkers have demonstrated for long chain alkanes a parallel orientation of the carbon skeleton with respect to HOPG surface [29]. Further, epitaxial growth of paraffin waxes on organic crystals also resulted in a parallel orientation of these waxes on organic crystals [30–31]. Additionally, the presence of free standing dangling bonds at the step edges of an HOPG substrate surface [32–33] could also influence the tubule orientation. The term epitaxial growth is however restricted to first few grown layers of crystal on the substrates. On the other hand, the tubules observed in our experiments were formed on an instantaneously solidified thin wax film (containing both tubule and non-tubule forming waxes) instead of directly on the substrate surface. At high wax concentration this thin film should effectively prevent the epitaxial relationship with HOPG. It is therefore surprising to see an upright orientation of tubules like on bare HOPG; thus, epitaxial relationship to the HOPG substrate does not seem to be the only essential criteria for upright growth of tubules.

At this point it is not surprising that shape and size of the tubules are independent of the concentration LWM_sol_ of their "building blocks" in the bulk solution, due to the very slow growth rate, which favors the inherent equilibrium structure of the wax objects, namely, tubules. On the other hand it remains unclear as to why they have a certain orientation, namely dominantly upright to the substrate surface, independent of the nominal thickness of an instantaneously solidified and therefore supposedly amorphous wax film, which, in turn, must be the reservoir for the following tubules' growth. One possibility would be a recrystallization of the initially formed amorphous film over time (Ensikat et al. have demonstrated a crystalline nature of wax deposits grown from wax material isolated from plants [34]) thus providing a template for epitaxial growth of tubules in an upright fashion. However, this is merely a speculation and no data is available to prove it.

Even at the rather low concentration of 0.2 mg/mL wax in solution tubules can still be formed on HOPG. It may be possible that tubules can still be formed on a HOPG substrate by further decreasing the concentration and it would be worth trying to determine the critical concentration needed at least for tubule formation on HOPG in particular in view of the equilibrium behavior expressed by Equation 1.

In the presence of water, the tubule completion time is reduced to almost half indicating a faster diffusion rate. This strongly supports the above described hypothesis of a higher molecular mobility in the presence of solvent remnants. Compared to chloroform, water molecules obviously enhance the diffusion rate even more and, hence, shorten the time period of tubule formation substantially. An increase in growth rate of such thin deposits in the presence of water has already been reported by various workers [35–39]. On hydrophobic surfaces water does not form ordered adsorption layers as the substrate–water interaction is believed to be considerably lower than the intramolecular hydrogen-bridge bonding interaction between the water molecules themselves although well-ordered structures of ice on HOPG are well documented [40]. On the other hand, a total internal reflection vibrational sum frequency spectroscopy study of the interaction of water molecules with molecules at a fluid hydrophobic surface, e.g., chloroform or hexane surfaces, by Scatena et al. [41] demonstrated a stronger electrostatic interaction between the organic surface and water than between the water molecules due to their intramolecular hydrogen-bridge bonding. This finding may also be applicable in our case. On the one hand, HOPG is hydrophobic, there is an existence of stronger interaction between the water molecules with both the chloroform and the long-chain organic wax molecules, which ultimately results in holding of the water (and remnant chloroform) molecules between wax molecules for a longer period of time. This presence of water (and chloroform) molecules appears to be responsible for the increased mobility of the wax molecules and, hence, the accelerated growth rate of the wax tubules.

No change in growth rate was found after adding salts to the chloroform, e.g., (NH_4_)_2_SO_4_ or NH_4_NO_3_. The average time period of "completion" of tubules is comparable to that in the absence of salts, i.e., in pure chloroform. Very few studies have been attributed to investigate the role of salts in the tubule growth [26,42]. However, these studies describe the influence of salts like nitrates on already formed wax crystals. On the other hand, our present study is to the best of our knowledge the first one using from the beginning salt containing solutions to understand the role of salts in the growth behavior of tubules. The lack of any change in the growth rate or orientation of the obtained tubules may suggest that salts like ammonium-nitrate or -sulfate do not seem to interact with wax molecules at all and, hence, have no effect on tubule growth rate or orientation. But a too low solubility of these two salts in chloroform (dipole moment 1.15 D) in order to expect any influence cannot be excluded. Therefore the tubule formation in the presence of salt and water was also studied and compared to that in the mere presence of water. In both cases completed tubules are formed within 2 h. As a result the addition of the salts does not have an observable influence on the growth rate of tubules, so that it is merely the added water alone that influences the growth, as already discussed above.

Since many years the transport of wax molecules from inside the plant surface cells to the outer side, where the wax crystals assemble, has been discussed. In vitro studies with isolated waxes and artificial membranes showed that the wax crystals dissolved when continuously in contact with water over several days on the one side, and re-crystallized on the other side of the membranes, suggesting that the water supports the transport through the (artificial) membranes [25]. Thus in natural systems it seems to be the water which plays a significant role in the transport of the wax components and their crystallization on the plant surface as well.

The growth behavior of tubules can be summarized in a schematic model as shown in Figure 2. The initial stage of growth are rodlets, these rodlets then grow either clockwise or anticlockwise (or occasionally simultaneously in both directions) on the surface until they form a ring structure. The ring structure finally grows upright in a spiral manner to form complete tubules.

The very high growth rate (before AFM imaging) may lead to the rodlets, while the following slower growth rate is characteristic for the subsequent tubule/height growth. The rodlet-growth requires only transport on the surface which is further aided by the presence of a greater number of solvent molecules in the initial stages of crystallization. As time passes through, solvent molecules start to evaporate thereby decreasing the rate of diffusion of wax molecules. Within the tubules, their growth is further hindered by the fact that incorporation of molecules above or below the tubules needs considerable activation energy which leads to a substantial decrease in their growth rate.

## Conclusion

The surface scientific approach presented here shows various factors affecting the growth characteristics of lotus wax tubules on a non-polar crystalline substrate like HOPG from chloroform based solutions. By recording consecutive AFM images of the re-crystallizing wax, we have studied the effect of a number of factors on the growth rate and the orientation of the tubules. By applying AFM imaging we show that different concentrations of wax molecules in pure chloroform have no effect on the growth rate of the tubules. The additional presence of water in the solution, however, considerably increases the growth rate but has no influence on the tubule orientation. On the other hand, the presence of the salts (NH_4_)_2_SO_4_ and NH_4_NO_3_, neither in pure chloroform nor in water saturated chloroform has any additional effect on the tubule growth or orientation at all. In future work we are extending our ideas to other substrates, e.g. mica, glassy carbon and glass, having similar or different properties than HOPG in order to find out the effect of these substrates and how the above mentioned factors influence the tubule growth on these substrates.

## Supporting Information

File 1 Video 1.
Lotus tubule growth on HOPG from 0.8 mg/mL chloroform wax solution. Only (forward) trace images are used for making videos. Size = 1*.*35 × 1*.*35 µm, scan rate = 0.796 Hz, 512 lines/image.

File 2 Video 2.
Lotus tubule growth on HOPG from 1 mg/mL chloroform wax solution. Only trace images are used for making videos. Size = 2.5 × 2.5 µm, scan rate = 0.512 Hz, 512 lines/image.

File 3 Video 3.
Lotus tubule growth on HOPG from 10 mg/mL chloroform wax solution. Only trace images are used for making videos. Size = 4.9 × 4.9 µm, scan rate = 0.512 Hz, 512 lines/image.

File 4 Video 4.
Lotus tubule growth on HOPG from 0.2 mg/mL chloroform wax solution. Only trace images are used for making videos. Size = 3.25 × 3.25 µm, scan rate = 0.519 Hz, 512 lines/image.

File 5 Video 5.
Lotus tubule growth on HOPG from 0.8 mg/mL water saturated chloroform wax solution. Only trace images are used for making videos. Size = 3 × 3 µm, scan rate = 1.04 Hz, 512 lines/image.

File 6 Video 6.
Lotus tubule growth on HOPG from 0.8 mg/mL (NH_4_)_2_SO_4_ salt saturated chloroform wax solution. Only trace images are used for making videos. Size = 1.2 × 1.2 µm, scan rate = 1.04 Hz, 512 lines/image.

File 7 Video 7.
Lotus tubule growth on HOPG from 0.8 mg/mL (NH_4_)_2_SO_4_/water saturated chloroform wax solution. Only trace images are used for making videos. Size = 2.05 × 2.05 µm, scan rate = 1.04 Hz, 512 lines/image.
